# Okadaic acid influences xenobiotic metabolism in HepaRG cells

**DOI:** 10.17179/excli2022-5033

**Published:** 2022-08-01

**Authors:** Leonie T.D. Wuerger, Helen S. Hammer, Ute Hofmann, Felicia Kudiabor, Holger Sieg, Albert Braeuning

**Affiliations:** 1 German Federal Institute for Risk Assessment, Department of Food Safety, Max-Dohrn-Str. 8-10, 10589 Berlin, Germany; 2 SIGNATOPE GmbH, Markwiesenstraße 55, 72770 Reutlingen, Germany; 3 Dr. Margarete Fischer-Bosch Institute of Clinical Pharmacology, Auerbachstr. 112, 70376 Stuttgart, and University of Tübingen, 72074 Tübingen, Germany

**Keywords:** okadaic acid, HepaRG cells, CYP enzymes

## Abstract

Okadaic acid (OA) is an algae-produced lipophilic marine biotoxin that accumulates in the fatty tissue of filter-feeding shellfish. Ingestion of contaminated shellfish leads to the diarrheic shellfish poisoning syndrome. Furthermore, several other effects of OA like genotoxicity, liver toxicity and tumor-promoting properties have been observed, probably linked to the phosphatase-inhibiting properties of the toxin. It has been shown that at high doses OA can disrupt the physical barrier of the intestinal epithelium. As the intestine and the liver do not only constitute a physical, but also a metabolic barrier against xenobiotic exposure, we here investigated the impact of OA on the expression of cytochrome P450 (CYP) enzymes and transporter proteins in human HepaRG cells liver cells *in vitro *at non-cytotoxic concentrations. The interplay of OA with known CYP inducers was also studied. Data show that the expression of various xenobiotic-metabolizing CYPs was downregulated after exposure to OA. Moreover, OA was able to counteract the activation of CYPs by their inducers. A number of transporters were also mainly downregulated. Overall, we demonstrate that OA has a significant effect on xenobiotic metabolism barrier in liver cells, highlighting the possibility for interactions of OA exposure with the metabolism of drugs and xenobiotics.

## Introduction

Okadaic acid (OA) is a marine biotoxin which is produced by dinoflagellates of the genus *Dinophysis* spp. and *Prorocentrum* spp. It is the lead compound of the group of diarrheic shellfish poisoning (DSP) toxins (EFSA, 2008[13]). OA is lipophilic and therefore able to accumulate in the fatty tissue of filter feeding organisms, for example shellfish. Ingestion of highly contaminated shellfish leads to DSP, which is characterized by severe gastrointestinal symptoms, including diarrhea, nausea, vomiting and abdominal pain. Therefore, the European Union has set a limit of 160 μg OA equivalents/kg shellfish meat (FAO, 2004[16]). With climate change, the occurrence of DSP toxin producing dinoflagellates is rising (Van Dolah, 2000[56]). OA has cytotoxic properties (Fessard et al., 1996[20]; Le Hégarat et al., 2006[35]; Ferron et al., 2014[19]), and apoptosis has been observed after exposure to OA in various mammalian cell lines (Bøe et al., 1991[6]; Lerga et al., 1999[37]; Morimoto et al., 1997[46]; Nuydens et al., 1998[47]). OA also leads to embryotoxic effects and may be able to pass the placental barrier (Ehlers et al., 2010[14]; Matias and Creppy, 1996[44]). The toxin acts as a tumor promoter in different organs in laboratory studies (Jiménez-Cárcamo et al., 2020[27]; Messner et al., 2006[45]) and it has been shown that people who frequently ingest OA-contaminated shellfish frequently are at higher risk of developing colon cancer (Cordier et al., 2000[8]; Manerio et al., 2008[42]; Lopez-Rodas et al., 2006[39]).

OA was originally isolated from the black sponge *Halichondria okadaic*. Its structure was first determined in 1981 (Tachibana et al., 1981[53]) and it was early identified as a protein phosphatase inhibitor. At the molecular level, OA mainly inhibits protein phosphatase 1 and 2A (Bialojan and Takai, 1988[5]), but also targets other serine/threonine phosphatases. Therefore, OA exposure leads to hyperphosphorylation of proteins, which in turn leads to changes in signal transduction and influences the cytoskeleton (Opsahl et al., 2013[48]). 

Because of the intestinal symptoms observed in humans after exposure to OA-contaminated seafood, previous studies on OA toxicity mainly focused on effects in the intestine. It is known, that OA can destroy the mechanic barrier of the intestine. Dietrich et al. demonstrated an effect of OA on the tight junction proteins, like claudins and occludin, at food-relevant concentrations, which may explain the disruption of the intestinal epithelium after exposure to OA (Dietrich et al., 2019[11]). As, however, OA does not only lead to mucosal (Dietrich et al., 2019[11]), but also to hepatic damage (Wang et al., 2021[57]), examining the hepatic effects of OA becomes more and more important with the rise in OA occurrence and more people being exposed. In addition to the physical barrier function provided by the intestinal epithelium, the intestinal enterocytes and especially the hepatocytes of the liver constitute a biochemical barrier, which decisively regulates oral bioavailability of drugs and xenobiotics via a battery of drug-metabolizing enzymes, transport proteins, and nuclear receptors providing an adaptive system of defence against xenobiotic exposure (Jetter and Kullak-Ublick, 2020[26]; Suzuki and Sugiyama, 2000[52]; Zanger and Schwab, 2013[60]). In contrast to the well-documented effects of OA on the physical intestinal barrier function, it has not yet been elucidated whether OA is also able to affect the biochemical barrier function of the liver and the intestine. This is of special importance as effects at sub-toxic doses would be able to interfere with the bioavailability of a plethora of drugs and xenobiotics.

With respect to the interaction of OA and xenobiotic metabolism, it has been documented that OA is oxidatively metabolized in human liver by CYP3A4 and CYP3A5, two cytochrome P450 (CYP) enzymes (Guo et al., 2010[23]; Kittler et al., 2014[29]). However, further interactions with or influences of OA on other CYP enzymes have not been elucidated. Therefore, the aim of this study was to identify the effects of exposure to okadaic acid on xenobiotic enzymes in human liver cells and thereby understanding the effects of OA on the metabolic barrier, particularly the CYPs and transporter proteins. We used HepaRG cells and incubated them with OA and different CYP-activating effectors. The HepaRG cell line originates from a human hepatocarcinoma and can be differentiated into hepatocyte-like and biliary epithelium-like cells. Differentiated HepaRG cells express a variety of liver-specific enzymes, especially enzymes of xenobiotic metabolism at levels close to primary hepatocytes and are therefore a frequently used model to examine human xenobiotic metabolism *in vitro *(Tascher et al., 2019[55]; Kanebratt and Andersson, 2008[28]). 

## Materials and Methods

### Chemicals

OA and 6-(4-chlorophenyl) imidazo[2,1-*b*][1,3]thiazole-5-carbaldehyde O-(3,4-dichlorobenzyl) oxime (CITCO) were purchased from Enzo Life Sciences GmbH (Loerrach, Germany). Benzo[k]fluoranthrene (BkF) was kindly provided by Dr. Albrecht Seidel (Biochemical Institute for Environmental Carcinogens, Prof. Dr. Gernot Grimmer Foundation, Großhansdorf, Germany). All other chemicals and materials were obtained from Sigma Aldrich (Taufkirchen, Germany) and Roth (Karlsruhe, Germany) in the highest available purity.

### Cell cultivation 

HepaRG cells were purchased from Biopredic International (Saint-Grégoire, France) and cultivated at 37 °C for 14 days in William's E medium supplemented with 10 % fetal bovine serum (FBS), 5 μg/mL insulin (medium and both supplements from PAN-Biotech GmbH, Aidenbach, Germany), 50 μM hydrocortisone hemisuccinate (Sigma Aldrich, Steinheim, Germany), 100 U/mL penicillin and 100 μg/mL streptomycin (Capricorn Scientific, Ebsdorfergrund, Germany), with a change of medium every 2-3 days. After 14 days, 1.7 % dimethyl sulfoxide (DMSO) was added to the medium for additional 14 days to induce differentiation of the HepaRG cells. The medium was then changed to serum-free assay medium (SFM) for two days. SFM was adapted from Klein et al. (2014[31]). It consists of William's E medium without phenol red (PAN-Biotech GmbH, Aidenbach, Germany) supplemented with 100 U/mL penicillin and 100 μg/mL streptomycin, 2.5 μM hydrocortisone hemisuccinate, 10 ng/mL human hepatocyte growth factor (Biomol GmbH, Hamburg, Germany), 2 ng/mL mouse epidermal growth factor (Sigma Aldrich, Steinheim, Germany) and 0.5 % DMSO. 

### Cell viability testing

To determine the optimal OA concentrations, cells were exposed to different OA concentrations and cell viability after 24 h was determined using the tetrazolium dye 3-(4,5-dimethylthiazol-2-yl)-2,5-diphenyltetrazolium bromide (MTT; Biomol GmbH, Hamburg, Germany). 9 × 10^3^ cells/well were seeded in 96-well plates and cells were cultivated as described in the section “Cell cultivation”. After differentiation, cells were incubated with different OA concentrations (5 nM - 500 nM). 0.01 % Triton-X in SFM was used as positive control for cytotoxicity. After 23 h of incubation, 10 µL MTT stock solution (5 mg/mL in phosphate-buffered saline) was added and incubated for an additional hour at 37 °C. The supernatant was then discarded and 130 µL desorption solution (0.7 % sodium dodecyl sulfate (SDS) diluted in 2-propanol) was added to each well. The plate was put on a shaker at 600 rpm for 30 minutes. Afterwards, the absorption of the generated formazan dye was determined at 570 nm using a Tecan infinite M200 PRO microplate reader (Tecan Group Ltd., Männedorf, Switzerland). The absorption values were offset against the reference wavelength at 630 nm. Incubation with the test substances occurred in SFM with 33 nM OA (MTT assay in Supplementary Figure 1), 5 µM BkF, 5 µM CITCO and 20 µM SR12813 for 24 h each.

### Gene expression analysis

The cells were seeded in 6-well plates with 2 × 10^5^ cells/well and treated as mentioned above. After incubation with OA, the gene expression of CYP enzymes and transport proteins was analyzed with quantitative real-time reverse transcriptase polymerase chain reaction (qPCR). For this purpose, cells were washed with ice-cold PBS and lysis was performed using ice-cold RLT buffer (RNeasy Mini Kit, Qiagen GmbH, Hilden, Germany) containing 1 % β-mercaptoethanol (Merck Schuchardt OHG, Hohenbrunn, Germany). RNA extraction was then performed following the instructions of the RNeasy Mini Kit (Qiagen GmbH, Hilden, Germany). The RNA was reversely transcribed into cDNA using the High Capacity cDNA Reverse Transcription Kit (Applied Biosystems, Foster City, USA). The qPCR was conducted using the LightCycler 96 instrument (Roche, Mannheim, Germany) with the Maxima SYBR Green/ROX qPCR Master Mix (Thermo Fisher Scientific, Waltham, USA). The temperature profile for the qPCR is shown in Supplementary Table 1. As housekeeping genes, the constitutively expressed genes glyceraldehyde 3-phosphate dehydrogenase (*GAPDH*) and β-glucuronidase *(GUSB)* were used. Analysis was done using the 2^-ΔΔct^ method (Livak and Schmittgen, 2001[38]), the samples were normalized to the geometric mean of the housekeeping genes. All primer sequences are summarized in Supplementary Table 2. They were synthesized by Eurofins (Hamburg, Germany).

### Protein quantification

Cells were seeded in 6-well plates and treated as described above; they were then washed twice with ice cold PBS and lysed with 500 µL lysis buffer (1 % NP-40, 0.01 % sodium dodecyl sulfate (SDS); 0.15 M sodium chloride; 0.01 M sodium phosphate; 2 mM ethylenediaminetetraacetic acid (EDTA); 2.5 U/ml benzonase; pH 7.2). Cell lysates were then incubated at 4 °C for 1 h on a rotor and afterwards snap-frozen in liquid nitrogen.

Protein quantification was conducted according to the methodology described in Weiß et al. (2018[59]). Briefly, prior to analysis, the total protein amount was determined via the bicinchoninic acid (BCA) assay (Thermo Fisher Scientific, Waltham, USA), and 150 µg protein was proteolyzed using trypsin (Pierce Trypsin Protease, MS-grade; Thermo Fisher Scientific, Waltham, USA). Surrogate peptides and internal standard peptides were precipitated using triple X proteomics (TXP) antibodies. Peptides were then eluted and quantified using a modified version of the previously described LC-MS methods (Braeuning et al., 2020[7]; Belghit, 2021[3]): There, 6 min, 12 min, and 20 min parallel reaction monitoring (PRM) methods are described. In the present project, the transporters, which had been analyzed with a 12 min gradient before, were transferred to a 10 min method, which is an elongated version of the 6 min method. The CYPs were analyzed with the 20 min method (UltiMate 3000 RSLCnano and PRM-QExactive Plus; Thermo Fisher Scientific, Waltham, USA). Raw data were processed using Skyline software (MACOSS Lab, Department of Genome Sciences, University of Washington, Seattle, USA) and TraceFinder 4.1 (Thermo Fisher Scientific, Waltham, USA). Peptide amounts were calculated by forming the ratios of the integrated peaks of the endogenous peptides and the isotope-labeled standards. Quantities of proteins were reported normalized as fmol per µg extracted protein.

### CYP activity assay

0.55 x 10^5^ cells/well were seeded in 24-well plates and treated as described in part 0. After 24 h of incubation with OA, the culture medium was replaced by SFM containing CYP substrates (50 µM phenacetin; 25 µM bupropion; 5 µM amodiaquine; 100 µM tolbutamide; 100 µM mephenytoin; 5 µM propafenone; 35 µM atorvastatin) and incubated at 37 °C for 3 h. Afterwards, formic acid was added to the medium with a final acid concentration of 50 mM to stop the reaction. Formation of metabolites was measured as previously described (Knebel et al., 2018[32]; Feidt et al., 2010[18]) and stood proportional to active enzyme levels. 

### Statistical analysis

Statistical analysis was performed using the software Sigma Plot for the expression of CYP enzymes at the mRNA and protein levels, and for the transporter protein expression. To determine statistical significance, one-way ANOVA followed by Dunnett's post-hoc test (*p < 0.05; **p < 0.01; ***p < 0.001) was used to compare the different sample groups. For the RNA expression of transporter proteins, where only one sample group was compared to the solvent control, Student's t-test was used to determine statistical significance.

## Results

### OA downregulates expression of CYP enzymes

To assess changes in xenobiotic liver metabolism after exposure to OA, a non-cytotoxic concentration of 33 nM OA was chosen as a non-cytotoxic concentration according to the results of cell viability testing (Supplementary Figure 1). Micrographs of the treated HepaRG cells can be found in Supplementary Figure 3. We first investigated the effects of OA on CYP enzymes, the most important family of phase I xenobiotic-metabolizing enzymes. Representative CYPs for different CYP families were chosen in addition to the CYPs which were induced by nuclear receptors. HepaRG cells were incubated with OA for 24 h. Cells were additionally treated, in the absence or presence of OA, with different effectors known to induce the expression of specific CYPs via activation of nuclear receptors (CITCO, BkF and SR12813) for 24 h. CITCO significantly activates the constitutive androstane receptor (CAR) and subsequently the transcription of CYPs especially from the CYP2B and CYP2C sub-families, BkF activates the aryl hydrocarbon receptor (AHR) and subsequently CYPs especially from sub-family CYP1A, while SR12813 is an activator of the pregnane-X-receptor (PXR) and its model target CYP3A4 (Mackowiak and Wang, 2016[41]; Goedtke et al., 2020[22]; Watkins et al., 2001[58]).

**Figure 1:**
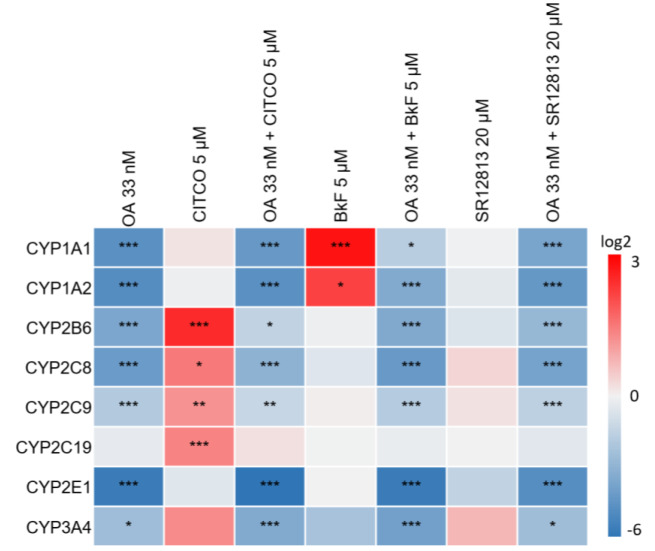
Effect of OA on cytochrome P450 (CYP) mRNA levels. Differentiated HepaRG cells were treated with 33 nM OA, alone or in combination with 5 µM CITCO, 5 µM BkF, 20 µM SR12813, or with the respective solvent. Analysis of mRNA levels was performed by qPCR. The heatmap shows the log_2_ values of the resulting fold changes as mean of three independent replicates, relative to the solvent control. Statistical analysis (n=3) was performed using one-way ANOVA followed by Dunnett's post-hoc test (*p < 0.05; **p < 0.01; ***p < 0.001).

As evident from Figure 1(Fig. 1), OA was able to downregulate a broad spectrum of CYPs at the mRNA level: significantly lowered mRNA levels were observed for CYP1A1, CYP1A2, CYP2B6, CYP2C8, CYP2C9, CYP2E1, and CYP3A4. Statistically significant induction of CYP2B and CYP2C isoforms was observed after treatment with CITCO, and this effect was antagonized by the presence of OA (Figure 1(Fig. 1)). Similarly, induction of CYP1A1 and CYP1A2 by BkF was antagonized by OA, and the inducing effect of SR12813 on CYP3A4, albeit not statistically significant in our analysis, disappeared when cells were additionally treated with OA (Figure 1(Fig. 1)). In summary, OA was able to diminish both, the basal expression of xenobiotic-metabolizing CYPs, as well as their induction by xenobiotic nuclear receptor agonists.

**Figure 2:**
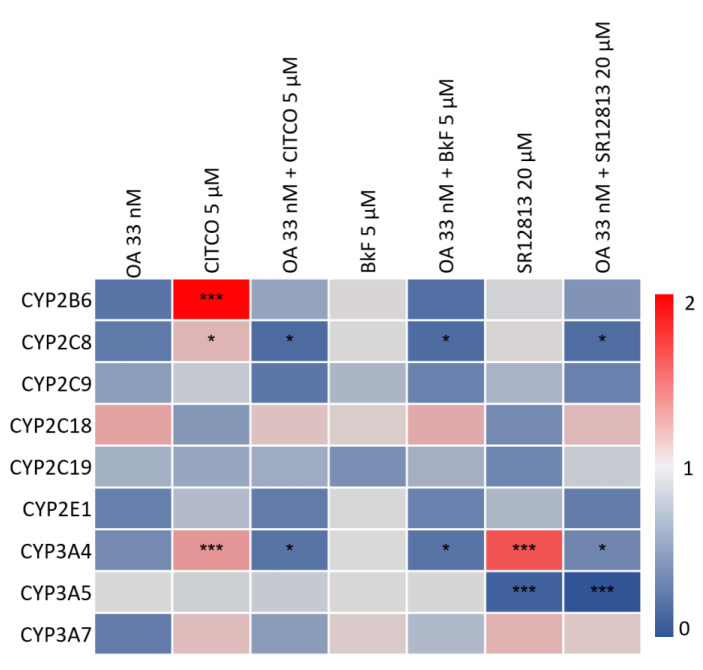
Effect of OA on CYP protein expression. HepaRG cells were treated as outlined in the legend to Figure 1. Cells were lysed and protein amounts were determined using the BCA assay. Proteins were proteolyzed using trypsin and analyzed using LC-MS. Raw data were processed using Skyline software and TraceFinder 4.1. Peptide amounts were calculated by forming the ratios of the integrated peaks of the endogenous peptides and the isotope-labeled standards. Samples were then normalized to the solvent-treated control. Statistical analysis was performed by one-way ANOVA followed by Dunnett's posthoc test (*p < 0.05; **p < 0.01; ***p < 0.001).

**Figure 3:**
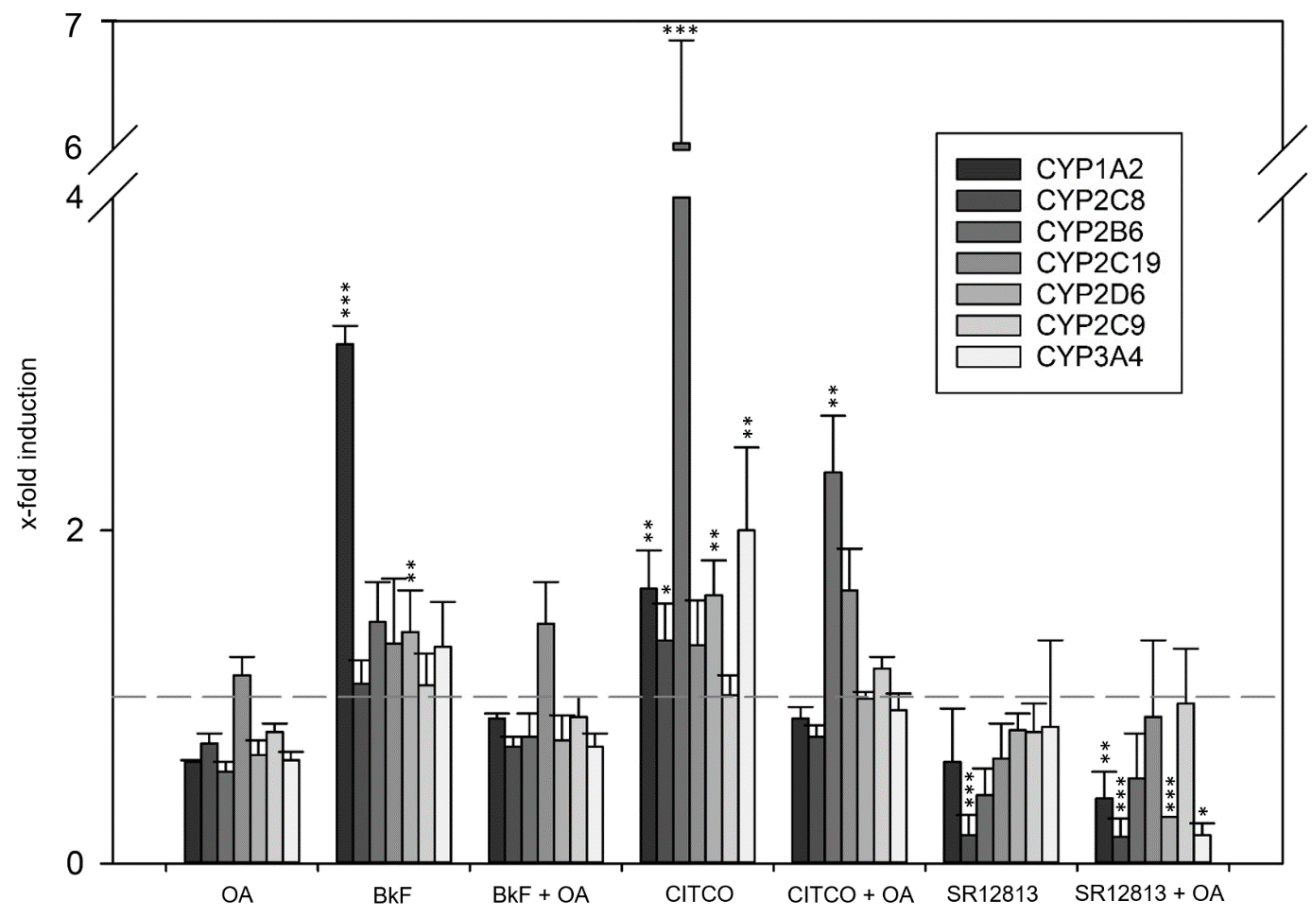
Activity of selected CYP enzymes. HepaRG cells were treated as outlined in the legend to Figure 1 and then incubated with CYP substrates for 3 h. Metabolite formation equal to the enzymatic activity of each CYP was determined using a mass-spectrometric approach. The results were normalized to the solvent-treated control. Statistical analysis (n=3) was performed by one-way ANOVA followed by Dunnett's post-hoc test (*p < 0.05; **p < 0.01; ***p < 0.001).

Data at the mRNA level were complemented by mass-spectrometric determination of selected CYPs at the protein level following an identical experimental approach for treatment with OA and CYP inducers. Comparable to what had been observed at the mRNA level, exposure of HepaRG cells to OA led to diminished levels of most CYPs (Figure 2(Fig. 2)). Statistically significant induction of CYP2B6 and CYP3A4 by CITCO, as well as of CYP3A4 by SR12813 was observed, and again counteracted by co-incubation of HepaRG cells with 33 nM OA (Figure 2(Fig. 2)). As a third layer of analysis, catalytic activities of CYP enzymes were measured. Comparable to the observations at the mRNA and protein levels, CYP activities were reduced by OA treatment, and the pronounced induction of CYP1A2 by BkF, and of CYP2B6 and CYP3A4 by CITCO was antagonized by simultaneous exposure to OA (Figure 3(Fig. 3)). Taken together, we detected a broad-spectrum inhibition of CYP expression and activity in Hepa-RG cells treated with a non-toxic concentration of OA.

### OA influences expression of transporter proteins in liver

**Figure 4:**
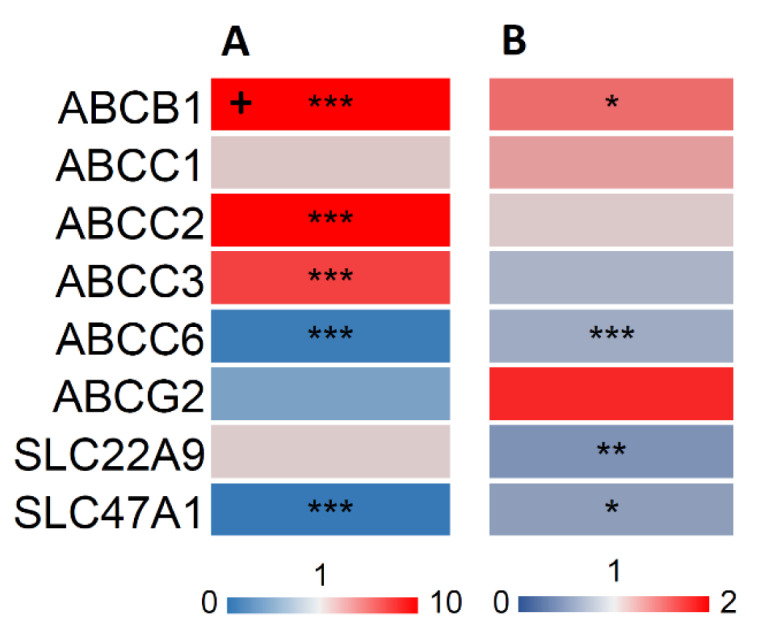
Effect of OA on transporter mRNA (A) and protein (B) levels. HepaRG cells were treated as outlined in the legend to Figure 1, and mRNA levels were determined by qPCR and normalized to the housekeeping genes GUSB and GAPDH, while protein levels were assessed using LC-MS and calculated relative to isotope-labeled standards. The heatmaps shows the resulting fold changes of three independent replicates. Marking with “+” indicates a fold change value outside of the color scheme (i.e. value >10). Statistical analysis was performed using one-way ANOVA followed by Dunnett's posthoc test (*p < 0.05; **p < 0.01; ***p < 0.001).

Besides metabolic enzymes such as the CYPs, transport proteins play a decisive role in the bodies' defense against foreign compounds. To assess the influence of OA on these proteins, we examined the mRNA and protein expression of a representative set of transporters from the ABC and SLC/SLCO families. ABCB1, ABCC2 and ABCC3 were significantly upregulated after exposure of HepaRG cells to OA for 24 h at the mRNA level, but only ABCB1 was significantly upregulated in the protein level (Figure 4(Fig. 4)). Furthermore, a significant downregulation of 2 transporters (ABCC6 and SLC47A1) at the mRNA level and of 3 transporters (ABCC6, SLC22A9 and SLC47A1) at the protein level was observed (Figure 4(Fig. 4)).

## Discussion

The ingestion of OA-contaminated shellfish leads to DSP, which is associated with severe gastrointestinal symptoms like nausea, vomiting and abdominal pain. OA producing dinoflagellates are especially abundant in European waters. Therefore, OA-contaminated shellfish is a remarkable health concern particularly in the EU. The current limit of 160 μg OA eq./kg shellfish was thus implemented in the EU in 2008 to protect consumers from OA toxicity (EFSA, 2008[13]). Since then, new insights into the effects of OA have emerged. For example, it has recently been shown that OA is able to pass the physical barrier of the intestine by affecting tight junction proteins (Dietrich et al., 2019[11]). Thereby, OA is also able to reach more remote organs, primarily in the liver. The long term effects of OA in the liver, especially after exposure to lower doses that do not induce DSP, have not been within the focus of scientific research so far. However, as regulation of OA levels in shellfish helps preventing acute poisoning, exposure of consumers to sub-toxic doses becomes more likely due to the rising occurrence of OA-producing organisms. This highlights the need for research in that field. We chose the hepatic biochemical barrier against xenobiotic exposure as subject of research, as it is well known that different drugs or xenobiotics are capable of remarkably affecting hepatic xenobiotic metabolism even at non-toxic doses. For example, ingestion of activators or inhibitors of drug-metabolizing enzymes can lead to unexpected mixture effects following combined exposure to chemicals, or be the cause for pharmacokinetics-based drug-drug interactions (Martin et al., 2021[43]; Hakkola et al., 2020[24]). Functionalization of xenobiotics constitutes the first phase of drug metabolism, with the CYP enzymes being the most important family of enzymes here. 57 different CYP enzymes have been identified in the human body, which facilitate different reactions (Zanger and Schwab, 2013[60]). The second phase is conjugating the xenobiotics with endogenous substances, which makes them more hydrophilic and enables them to be transported and excreted in phase III of xenobiotic metabolism through specific transporters like ABC or SLC transporters. Our results were obtained in HepaRG cells, a human cell line that differentiates into hepatocyte-like cells. It has been shown that HepaRG cells express CYP enzymes and other important enzymes of xenobiotic metabolism and that they are therefore a well-suited model to investigate the regulation and activity of xenobiotic metabolism *in vitro* (Kanebratt and Andersson, 2008[28]; Tascher et al., 2019[55]).

To the best of our knowledge, there has not been a study concerning the direct effects of OA on CYP enzyme expression and activity and transporter protein expression in human cells so far. Published studies in the context of OA and xenobiotic metabolism mainly focused on the metabolism of OA itself (Kolrep et al., 2016[33]). It is known that OA is metabolized and detoxified by CYP3A4 and CYP3A5 (Kittler et al., 2014[29]; Guo et al., 2010[23]) and gets bioactivated by CYP1A2 (Kolrep et al., 2016[33]). Our findings show that OA, or possibly also a metabolite of OA formed inside the cells, is able to inhibit the expression of several CYP enzymes and also to inhibit their metabolic activity. In additional experiments, we used nuclear receptor agonists to increase CYP levels. Our findings show that OA is able to counteract the effects of these CYP inducers. Moreover, except for ABCB1, ABCC2 and ABCC3 we observed reduced expression of transporters following incubation of cells with OA. ABCB1, ABCC2 and ABCC3 were upregulated on mRNA - but only ABCB1 also on protein level. ABCB1 is also called multi-drug resistance protein 1 (MDR1). Elevated ABCB1 levels indicate a role of this protein in cellular elimination of OA, which has been described before (Dietrich et al., 2020[12]; Ehlers et al., 2014[15]). Upregulation on RNA but not on protein level indicates a possible overlaying mechanism on the protein translation. ABCG2 is downregulated on the RNA but upregulated on the protein level. It has been previously described as upregulated by OA (Dietrich et al., 2020[12]). ABC proteins are generally very stable, and are also intracellularly stored and recycled (Ben Saad et al., 2021[4]; Czuba et al., 2018[9]). Therefore it is not surprising to detect a low RNA expression while still having a high amount of ABCG2 present in the cell.

The observation that OA is able to down-regulate expression of such a broad spectrum of drug-metabolizing enzymes and transport proteins rather points towards an effect of OA via a general mechanism, than to effects based on individual gene- or protein-specific mechanisms. This is supported by the downregulation of several transcription factors in HepaRG cells after exposure to OA, which is shown in Supplementary Figure 2. There is evidence that the activity of ABC transporters is modulated by phosphorylation (Stolarczyk et al., 2011[51]; Czuba et al., 2018[9]). SLC transporters are also regulated through phosphorylation and also through Protein kinase C (Czuba et al., 2018[9]), whose activity is also heavily influenced by phosphatases (Gao et al., 2008[21]). As a phosphatase inhibitor, it is likely, that OA can interfere in the phosphorylation of the transporters, thereby influencing their activity. These mechanisms, however, cannot explain the downregulation of transcript levels, as observed not only for various transporters, but especially also for most of the CYP genes analyzed. Instead, it is tempting to speculate that a more superordinate type of regulator is affected by OA, thereby impacting the expression of CYPs and transporters. Previous work has, for example, highlighted that transcription factors from the hepatocyte nuclear factor family are capable of affecting the expression of a broad spectrum of hepatocyte-specific genes, and also that the canonical Wnt/β-catenin signaling pathway has a strong impact on hepatic CYP expression (Lau et al., 2018[34]; MacDonald et al., 2009[40]). As β-catenin, however, appears to have an inducing effect on many CYPs and as we have previously shown activation of β-catenin by OA in human cells *in vitro*, a direct role of β-catenin signaling in CYP downregulation by OA does not appear likely. Another potential general mechanisms by which OA influences the expression of drug-metabolizing enzymes might be related to the activation of inflammation-related signaling: previous work has demonstrated that different interleukins have inhibitory effects on CYP and transporter expression in HepaRG cells (Tanner et al., 2018[54]; Febvre-James et al., 2018[17]; Le Vée et al., 2020[36]; Klein et al., 2015[30]), while OA is able to modulate the release of inflammatory cytokines in intestinal and immune cells (Del Campo et al., 2017[10]; Alarcan et al., 2019[1]).

Humans exposed to OA are commonly at the same time exposed to other chemical entities as well, such as food components, toxins and contaminants. Substances occurring in grapefruits, oranges and different herbs are known to influence the expression of xenobiotic-metabolizing enzymes and thereby constitute a possible risk for unintended pharmaco-/toxicokinetic effects in the consumers (Hanley et al., 2011[25]; Petric et al., 2020[49]; Saxena et al., 2008[50]). With an inhibitory effect on several CYP enzymes and also on their metabolic activity, the ingestion of OA may provoke similar effects in a scenario of combined exposure to other chemicals or drugs that are metabolized by the enzymes affected by OA. Furthermore, the downregulation of certain transport proteins may contribute to increased intracellular levels of certain xenobiotics or their metabolites, which might be of concern for example in case of co-exposure to different phycotoxins (Alarcan et al., 2018[2]). Future studies concerning the toxicological effects of mixture of OA with other substances will be required to fully understand the impact of OA on the kinetics of other xenobiotics, and the possible risks arising from such interaction.

Taken together, we have demonstrated that OA is able to decrease the mRNA expression and activity of several xenobiotic-metabolizing CYP enzymes in HepaRG cells. OA is furthermore able to counteract the effects of the CYP activators CITCO, BkF and SR12813 on these enzymes. We also detected an OA-induced change in transporter mRNA and protein expression. In conclusion, we show that OA is not only able to disturb the physical barrier function of the intestine, but also able to interfere with enzymes and proteins which are important for the biochemical metabolic barrier function of the liver. The present observations thus add an important aspect to the toxicological profile of OA.

## Declaration

### Author contributions 

Leonie T.D. Wuerger 1,2,3,4

Helen S. Hammer 1,2

Ute Hofmann 1,2

Felicia Kudiabor 1

Holger Sieg 2,3,4

Albert Braeuning 3,4,5

1. Performed experiments
2. Data evaluation
3. Writing the manuscript
4. Project planning
5. Supervision and funding

### Declaration of competing interest

We declare that there is no competing interest.

### Acknowledgment

UH was supported by the Robert Bosch Stiftung, Stuttgart. Furthermore, the authors would like to thank Annika Nielsen for providing laboratory assistance. This project was funded by the German Federal Institute for Risk Assessment, Germany (project 1322-766).

## Supplementary Material

Supplementary information
