# Supplementary material for: Okadaic acid influences xenobiotic metabolism in HepaRG cells
Source: EXCLI J. 2022 Aug 1;21:1053–65. doi: 10.17179/excli2022-5033 (PMC9489895; doi:10.17179/excli2022-5033)
Supplement: Supplementary information [file EXCLI-21-1053-s-001.pdf]

EXCLI Journal 2022;21:1053-1065 – ISSN 1611-2156
Received: May 11, 2022, accepted: July 28, 2022, published: August 01, 2022

Supplementary information to:

Original article:

OKADAIC ACID INFLUENCES XENOBIOTIC METABOLISM IN HEPARG CELLS

Leonie T.D. Wuerger[a], Helen S. Hammer[b], Ute Hofmann[c], Felicia Kudiabor[a], Holger Sieg[a]*, Albert Braeuning[a]

[a] German Federal Institute for Risk Assessment, Department of Food Safety, Max-Dohrn-Str. 8-10, 10589 Berlin, Germany
[b] SIGNATOPE GmbH, Markwiesenstraße 55, 72770 Reutlingen, Germany
[c] Dr. Margarete Fischer-Bosch Institute of Clinical Pharmacology, Auerbachstr. 112, 70376 Stuttgart, and University of Tübingen, 72074 Tübingen, Germany

* **Corresponding author:** Holger Sieg, German Federal Institute for Risk Assessment, Department of Food Safety, Max-Dohrn-Str. 8-10, 10589 Berlin, Germany, E-mail: holger.sieg@bfr.bund.de

https://dx.doi.org/10.17179/excli2022-5033

This is an Open Access article distributed under the terms of the Creative Commons Attribution License (http://creativecommons.org/licenses/by/4.0/).

EXCLI Journal 2022;21:1053-1065 – ISSN 1611-2156
Received: May 11, 2022, accepted: July 28, 2022, published: August 01, 2022

Supplementary Table 1: Temperature profile used for the qPCR

Reaction	Temperature	Time	Repeats
**Initialization**	50°C<br>95°C	2 min<br>10 min	1
**Denaturation, primer binding and elonga-tion**	95°C<br>60°C<br>72°C	10 s<br>15 s<br>20 s	40
**Dissociation curve**	95°C<br>60°C<br>95°C	15s<br>15s<br>15s	1

Supplementary Table 2: Used primer sequences for the qPCR

Protein name	Forward primer	Reverse primer
**CYP1A1**	ACCCTGAAGGTGACAGTTCC	TCTTGGAGGTGGCTGAGGTA
**CYP1A2**	AATCAGGTGGTGGTGTCAGT	TGAGTGTTCTTCACGAGGCT
**CYP2B6**	TTCGGCGATTCTCTGTGACC	ATGAGGGCCCCCTTGGAT
**CYP2C8**	CCTCTCATCAAATCTTCCCATT	GCAGTGACCTGAACAACTCTCC
**CYP2C9**	AAATGGAGAAGGAAAAGCACAACC	TCAACTGCAGTGTTTTCCAAGC
**CYP2C19**	CCTGGAACGCATGGTGGT	TCCATTGCTGAAAACGATTCCAAAT
**CYP2E1**	CATGAGATTCAGCGGTTCATC	GGTGTCTCGGGTTGCTTCA
**CYP3A4**	TCACAAACCGGAGGCCTTTT	TGGTGAAGGTTGGAGACAGC
**CYP7A1**	GACACACCTCGTGGTCCTCT	TTTCATTGCTTCTGGGTTCC
**GUSB**	CACCAGGGACCATCCAATAC	ATGTAGGTGGTGGGTGTCGT
**GAPDH**	TTAAAAGCAGCCCTGGTGAC	CTCTGCTCCTCCTGTTCGAC

EXCLI Journal 2022;21:1053-1065 – ISSN 1611-2156
Received: May 11, 2022, accepted: July 28, 2022, published: August 01, 2022

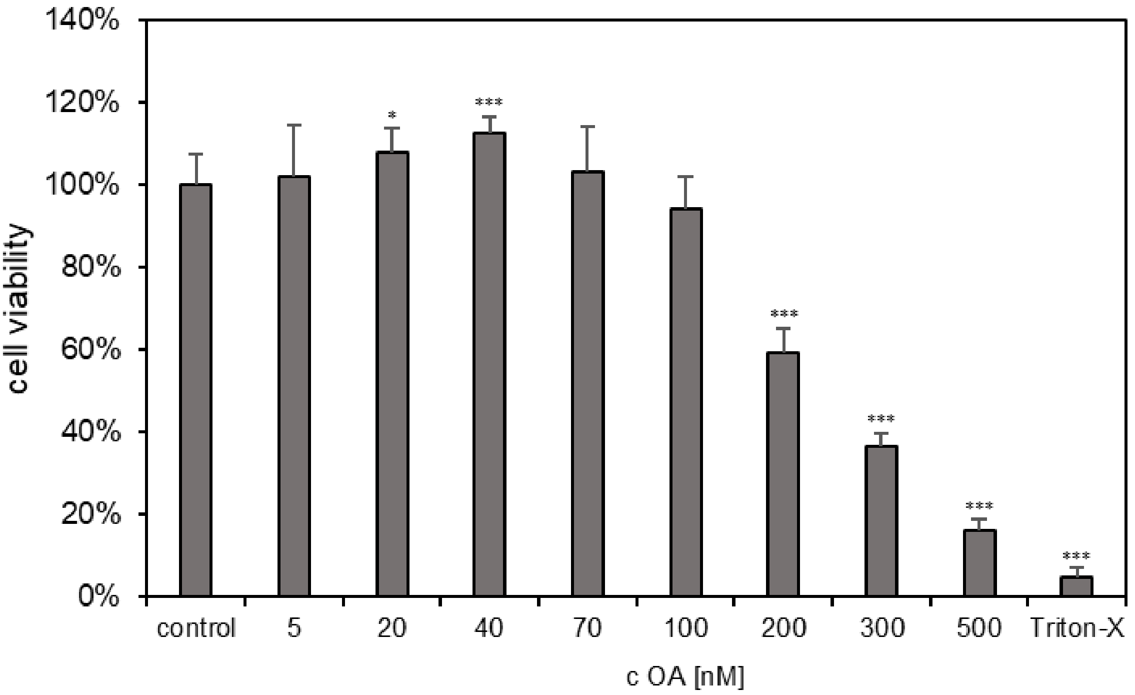

Supplementary Figure 1: MTT-Assay of selected OA concentrations on HepaRG cells. Cells were seeded in 96-well plates and grown for 2 weeks. They were then differentiated for 2 weeks prior to 24 h incubation. Cell viability is proportional to absorption at 570 nm and was compared to an untreated control. Based on the MTT assay, 100 nM OA was picked as the highest concentration for incubation. 11 and 33 nM were picked as non-toxic concentrations based on the 100 nM. Statistical analysis was performed using t-test (*p < 0.05; **p < 0.01; ***p < 0.001).

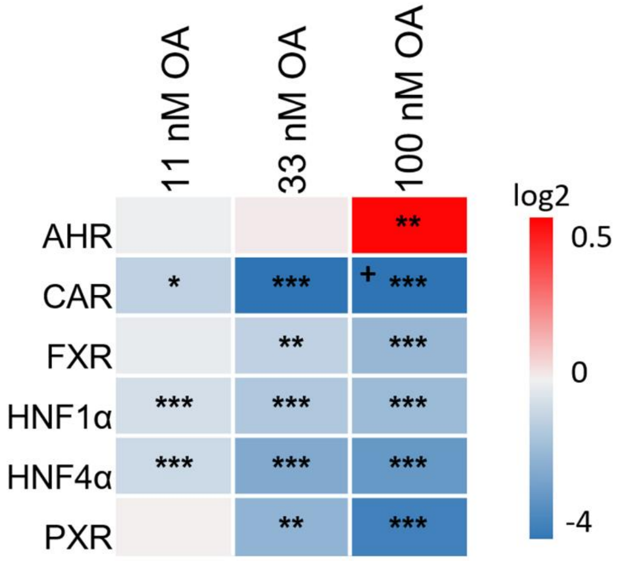

Supplementary Figure 2: Effect of OA on relevant transcription factors. HepaRG cells were treated with 11 nM, 33 nM and 100 nM OA, and mRNA levels were determined by qPCR and normalized to the housekeeping genes GUSB and GAPDH. The heatmap shows the log2 values of the resulting fold changes as mean of three independent replicates, relative to the solvent control. Marking with "+" indicates a fold change value outside of the color scheme (i.e. value <-4). Statistical analysis (=3) was performed using one-way ANOVA followed by Dunnett's post-hoc test (*p < 0.05; **p < 0.01; ***p < 0.001).

EXCLI Journal 2022;21:1053-1065 – ISSN 1611-2156
Received: May 11, 2022, accepted: July 28, 2022, published: August 01, 2022

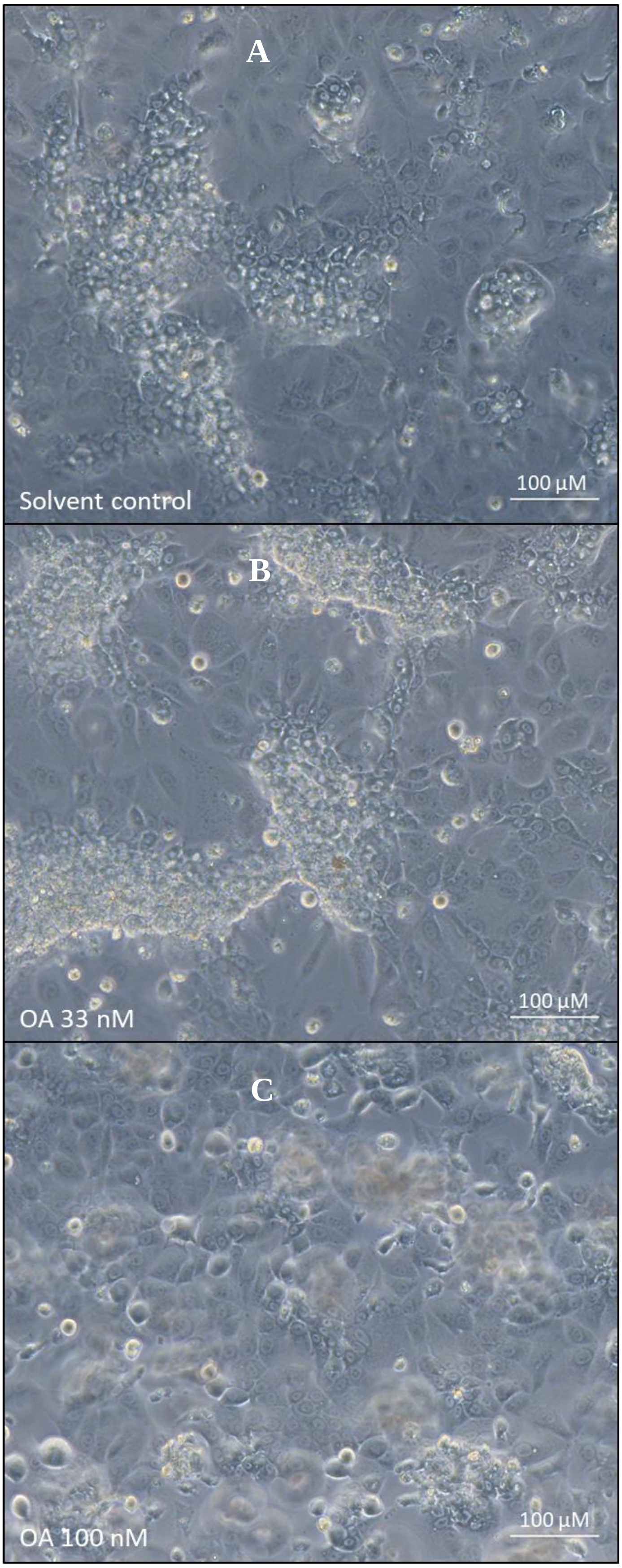

Supplementary Figure 3: Series of micrographs of the used HepaRG cells. Cells were were seeded in 12-well plates and grown for 2 weeks. They were then differentiated for 2 weeks prior to 24 h incubation. Cells were visualized using the Zeiss Axio Observer (Zeiss, Göttingen) and the Software ZEN 2.3 Pro for editing. **A** shows differentiated, untreated HepaRG cells after 2 weeks differentiation. **B** shows differentiated HepaRG cells incubated with 33 nM OA for 24 h. **C** shows differentiated HepaRG cells incubated with 100 nM OA for 24 h.
